# Psychological Resilience Is Associated With Participation Outcomes Following Mild to Severe Traumatic Brain Injury

**DOI:** 10.3389/fneur.2018.00563

**Published:** 2018-07-16

**Authors:** Carla Wardlaw, Amelia J. Hicks, Mark Sherer, Jennie L. Ponsford

**Affiliations:** ^1^Monash-Epworth Rehabilitation Research Centre, Monash Institute of Cognitive and Clinical Neurosciences, Monash University, Melbourne, VIC, Australia; ^2^TIRR Memorial Hermann, Houston, TX, United States; ^3^Department of Physical Medicine and Rehabilitation, Baylor College of Medicine, Houston, TX, United States

**Keywords:** traumatic brain injury, TBI, resilience, participation, depression, anxiety

## Abstract

Traumatic brain injury (TBI) causes physical and cognitive-behavioral impairments that reduce participation in employment, leisure, and social relationships. Demographic and injury-related factors account for a small proportion of variance in participation post-injury. Personal factors such as resilience may also impact outcomes. This study aimed to examine the association of resilience alongside demographic, injury-related, cognitive, emotional, and family factors with participation following TBI. It was hypothesized that resilience would make an independent contribution to participation outcomes after TBI. Participants included 245 individuals with mild-severe TBI [*M*_*age*_ = 44.41*, SD*_*age*_ = 16.09; post traumatic amnesia (PTA) duration *M* 24.95 days, *SD* 45.99] who completed the Participation Assessment with Recombined Tools-Objective (PART-O), TBI Quality of Life Resilience scale, Family Assessment Device General Functioning Scale, Rey Auditory Verbal Learning Test, National Adult Reading Test, and Hospital Anxiety and Depression Scale an average 4.63 years post-injury (*SD* 3.02, *R* 0.5–13). Multiple regression analyses were used to examine predictors of PART-O scores as the participation measure. Variables in the model accounted for a significant 38% of the variability in participation outcomes, *F*_(13, 211)_ = 9.93, *p* < 0.05, *R*^2^ = 0.38, adjusted *R*^2^ = 0.34. Resilience was a significant predictor of higher participation, along with shorter PTA duration, more years since injury, higher education and IQ, and younger age. Mediation analyses revealed depression mediated the relationship between resilience and participation. As greater resilience may protect against depression and enhance participation this may be a focus of intervention.

## Introduction

Following traumatic brain injury (TBI), participation in employment, education, leisure, and relationships is often significantly reduced, leaving individuals substantially less integrated in their communities (1–4). As a result, many individuals spend increased time at home, straining family and other relationships (5). Given that TBI occurs commonly during young adulthood (6), participation deficits coincide with a critical period of development in which individuals are completing education, establishing a vocation, leaving home, and forming important lifelong relationships. Failure to attain these goals may profoundly impact their sense of self, mental health and general well-being. Reduced participation often extends beyond the acute recovery period and continues to be associated with poorer quality of life up to two decades after injury (7). Arguably participation in these life roles, including employment, education, leisure and relationships, represents one of the most important and objective indicators of injury outcomes.

Numerous variables have been associated with participation outcomes post-TBI, including injury-related and demographic variables as well as post-injury environmental and personal factors. Injury severity, cognitive difficulties, and limb injuries with related pain and impact on mood, affect an individual's ability to engage socially and often present significant barriers to education and employment (8–16). Injury severity is a particularly well-researched predictor of participation outcomes, with duration of post traumatic amnesia (PTA) having the most robust association (17–21). With respect to demographic factors, younger age, higher premorbid education level, higher premorbid IQ, and being employed prior to injury have all been associated with better participation outcomes (10, 22–29). Notably, older age at injury has been found to predict both worse participation overall as well as progressively worsening participation over time (10). Although gender does not appear to be directly associated with participation (30), it may have an indirect association, for example through mood and pre-injury education (14). Post-injury psychological functioning, particularly depression and anxiety, are also important predictors of participation outcomes (10, 12, 31–33). The impact of family functioning on participation is thought to be both direct, and through association with emotional well-being (34, 35).

Due to this broad range of factors influencing outcome, research has moved toward a multivariate approach to prediction of participation outcomes following TBI (24, 36, 37, 38). These models contribute to a more comprehensive understanding of participation outcomes; however, the average amount of variance accounted for by predictive models is around 30% (21). This suggests there are additional predictive factors yet to be identified. One such factor that has increasingly gained scholarly recognition, due its positive association with quality of life and well-being outcomes among different clinical populations, is resilience.

Resilience has been conceptualized as a process of adaptation to adversity or the ability to bounce back after trauma or adversity. Resilience arguably influences the extent to which a person is able to resume important life roles after an injury. Resilience may impact participation outcomes directly through facilitating or promoting return to normal life or the development and achievement of new life goals (39), and indirectly through its effects on improved well-being, quality of life and psychological adjustment. Participating in employment, education, leisure, and relationships represent fundamental areas of participation. Resilience has been positively associated with physical and emotional well-being in individuals with cancer (40), Parkinson's disease (41), diabetes (42), chronic spinal cord injury (43), multiple sclerosis, spina bifida, stroke, and posttraumatic stress disorder (44, 45). There has been less resilience research in TBI, with only one study to date examining the association between resilience and participation. Notably, it has been suggested that the study of resilience after TBI poses a distinct challenge, in that the skills characteristically associated with resilience are typically impaired after TBI (45–47). For example, resilience requires emotional stability, a positive outlook, good problem-solving skills and social perception (47); however, TBI is commonly associated with impaired executive functioning (48, 49), irritability and aggression (50, 51), depression (33, 45), and difficulties with social perception (52).

The little research that has focused on resilience after TBI has been largely limited to patients with mild TBI, in whom no studies have examined impact on participation. In this group, greater resilience has been associated with less reporting of post-concussional and post-traumatic stress symptoms (53–55), reduced fatigue, insomnia, stress, and depressive symptoms, as well as better quality of life (56). One study found that greater pre-injury resilience was significantly associated with greater post-concussion symptom severity 1 month post-injury (57), perhaps reflecting insufficient time for participants to “bounce back” (44), or overrating of pre-injury resilience levels, a phenomenon known as the “Good Old Days”(58).

Only three studies have examined resilience in individuals with moderate to severe TBI, of which one examined an association with participation. Marwitz et al. (39), conducted a large (*n* = 195) longitudinal study and found that resilience was significantly associated with participation over the first 12 months post-injury (39). Other studies have associated higher resilience in individuals with moderate to severe TBI with fewer depressive and anxiety symptoms, better emotional adjustment, use of task oriented coping and greater social support (44, 45). However, one of these studies used a sample of individuals who were actively seeking help with adjusting to changes post-injury, possibly biasing the sample toward those experiencing greater adjustment problems (45).

The aim of the present study was to examine the relative association of resilience, as well as demographic, injury-related, cognitive, emotional, and family factors with participation (productivity, social relations and leisure) following mild to severe TBI. To the best of our knowledge, this is the first study to examine the association between resilience and participation outcomes more than 12 months after mild to severe TBI. This critically extends previous research by examining the impact of resilience across the spectrum of TBI severity, from mild to severe, and how this association influences outcomes beyond the acute post-injury period. It was hypothesized that resilience would make an independent contribution to participation after TBI, in a model that would include demographic variables (gender, age, pre-morbid IQ, education, pre-injury employment), injury variables (injury severity, cognitive functioning, limb injury, time since injury) and post-injury personal and environmental factors (depression, anxiety, family support).

## Materials and methods

This research was approved by the Epworth Human Research Ethics Committee and Monash University Human Research Ethics Committee. All participants gave written informed consent in accordance with the Declaration of Helsinki.

### Participants

The sample for this study was drawn from a larger prospective longitudinal head injury outcome study conducted at Epworth Hospital in Melbourne, Australia. Inclusion criteria for the current study included being aged 16 years or over and having a history of TBI sustained at least 3 months previously. Exclusion criteria included inadequate English or cognitive capability to complete the study measures, other pre-injury or post-injury neurological conditions or severe psychiatric disturbance (e.g., psychosis). Two-hundred and forty-five individuals met the eligibility criteria and consented to the study. There were no significant differences in age, gender, duration of PTA or GCS score between the study sample and patients who were admitted to the Epworth Hospital for rehabilitation and included in the longitudinal study during the same period (May 2004–July 2016). However, there was a significant difference in total years of education, with study participants having greater years of education (M 13.52 SD 3.09) compared to non-participants [*M* 10.24 *SD* 4.14; *t*_(386)_ = −8.7, *p* < 0.001].

As shown in Table 1, 73.5% of the sample were males (*M* = 43.50 years, *SD* = 15.55 years) and 26.5% were females (*M* = 46.95 years, *SD* = 17.25 years). The mean age of participants was 44.41 years (*SD* = 16.09, *R* = 17–78 years), and the majority of participants had sustained a severe TBI, based on PTA duration (*M* = 24.95 days, *SD* = 45.99; *R* = 0–455) and GCS score (*M* = 9.82, *SD* = 4.25; *R* = 3–15).

**Table 1:** Demographic, injury, personal, and environmental characteristics of participants with traumatic brain injury (*n* = 245).

**Variable**	***N***	***M***	***SD***	**Range**
Age at interview	245	44.41	16.09	17–78
Age at injury	245	40.16	16.48	16–77
Education (years)	245	13.52	3.09	6–27
Estimated FSIQ	239	109.02	7.53	88–127
Time since injury (years)	245	4.63	3.02	0.05–13
GCS	231	9.82	4.25	3–15
Mild (13–15)		42%		
Moderate (9–12)		16.9%		
Severe (3–8)		41.1%		
Duration of PTA (days)	234	24.95	45.99	0–455
<7 days		7.3%		
7–28 days		17.9%		
>28 days		74.8%		
Gender	245			
Male	180	73.5%		
Female	65	26.5%		
Employed before Injury	245			
Yes	217	88.6%		
No	28	11.4%		
Limb injury	244			
None	91	37.4%		
Minor	25	10.2%		
Moderate	63	25.8%		
Major	65	26.6%		

*FSIQ, Full Scale IQ; GCS, Glasgow Coma Scale; PTA, Post traumatic Amnesia*.

### Measures and procedures

Participants from the longitudinal head injury outcome study database were telephoned and invited to complete research interviews for the present study, a collaboration with Sherer et al. from TIRR Memorial Hermann, Houston, Texas, USA, identifying predictive models of TBI outcome (59). Recruitment and interviews occurred between January 2015 and June 2017. Participants were seen in their homes (66.9%) or at the hospital (33.1%) and were reimbursed for their time. The 90-min assessments included measures of mood, lifestyle and participation, and several cognitive measures. Demographic and injury data including gender, age, years of education, pre-injury employment, GCS scores, duration of PTA, and limb injuries were obtained from medical records and interviews.

#### National adult reading test

National Adult Reading Test (NART) (60). The NART consists of a 50-item word list, which the participant reads aloud. It is a validated as a measure of premorbid intellectual functioning in individuals post TBI (61).

#### Rey auditory verbal learning test

Rey Auditory Verbal Learning Test (RAVLT) (62). The RAVLT is a list learning memory task (40). The total words recalled for the five learning trials (RAVLT Trials 1-5) was used, as this has been identified as the most reliable measure (test-retest *r* = 0.77) (63). T-scores were generated (62), with higher scores reflecting better cognitive performance. The RAVLT is sensitive to the cognitive effects of TBI (49, 64).

#### Hospital anxiety and depression scale

Hospital Anxiety and Depression Scale (HADS) (65). The HADS comprises two subscales measuring anxiety and depression. Higher scores are indicative of higher depression and/or anxiety symptoms. The HADS has good internal consistency [Cronbach's α = 0.83 anxiety; α = 0.82 depression; (66)], and has been found to be a reliable and valid measure of emotional distress in TBI populations (67, 68).

#### Family assessment device general functioning scale

Family Assessment Device General Functioning Scale (FAD-12) (69). The FAD-12 is a 12-item subscale of the FAD, recommended for use as an index of family functioning (70). The higher the score, the more problematic the participant perceives the overall family functioning (71). The FAD-12 has good psychometric properties [Cronbach's α = 0.90; (72)], and has been validated for use in TBI populations (73, 74).

#### Traumatic brain injury quality of life resilience scale

Traumatic Brain Injury Quality of Life Resilience scale (TBI-QoL Resilience) (75). The TBI-QoL Resilience subscale is one of twenty subscales from the TBI Quality of Life measure. The 10-item measure uses a 5 point Likert scale and the total resilience score represents the individual's standing compared to that of other individuals with TBI (75). There has been limited analysis of the psychometrics of this scale, however, in a sample of military service members with mild TBI, the internal consistency was high [Cronbach's α = 0.91; (76)]. It has been suggested that the psychometric properties for the scale are likely to be strong due to the method of validation of the measure (77), which included focus groups, interviews, and patient consultation from individuals with TBI, clinicians, and caregivers of individuals with TBI. Additionally, item pools were tested in a large sample (*n* = 675) and calibrated using item response theory methods.

#### Participation assessment with recombined tools-objective

Participation Assessment with Recombined Tools-Objective [PART-O-17; (78)]. The PART-O measures frequency of productivity, “out and about” (e.g., going to the movies) and social relations, with higher scores indicative of greater community participation. The PART-O has been shown to have good construct and concurrent validity and the ability to reliably measure significant differences among individuals with varying levels of participation (79). The Averaged Total Score was used as an indication of overall participation post-injury (1). The PART-O has been shown to be an acceptable measure of participation for individuals with moderate and severe TBI (79) and is recommended for assessing social role participation in the TBI population by the National Institute of Neurological Disorders and Stroke (80).

### Data analysis

A multiple regression analysis using SPSSv.24 (SPSS, Inc., Chicago, IL) was undertaken to assess the extent to which selected variables predicted participation outcomes on the PART-O. Listwise deletion was deemed appropriate for all analyses as the total missing data represented <0.3% of responses and was judged to be missing completely at random (MCAR; Little's MCAR *p* > 0.05) (81–83). Sample size requirements for a multiple regression with 13 predictors were met (83). Five univariate outliers were identified (*z* score ± 3.29 standard deviations from the mean) (83) but found to be valid clinical cases meeting study inclusion criteria. Furthermore, standardized residuals statistics showed no residuals that were ± 3.29 standard deviations from the mean, and Cook's Distance had a maximum value of 0.44 (84), indicating that there were likely no cases having an undue influence on the regression model. Assumptions of normality of the dependent variable, multicollinearity, normality, linearity and homoscedasticity of residuals and independence of errors were all met. There was no evidence of multicollinearity: all tolerance values were > 0.10 and all variance inflation factors were <10 (85). Furthermore, individual examination of the correlation values between independent variables showed none above the 0.80 threshold (85).

Due to extensive research demonstrating PTA to be a more robust reflection of TBI severity and predictor of outcomes (14, 19, 86), PTA rather than GCS was included in the regression model. The following predictor variables were regressed on the outcome variable (PART-O): TBI QoL resilience, age at interview, gender, total years of education, employment status pre-injury, PTA duration, limb injury, premorbid IQ, RAVLT Trials 1-5 score, FAD-12 score, HADS anxiety score, HADS depression score, and years since injury.

Mediation analyses were conducted to explore whether depression and anxiety symptoms mediated the relationship between resilience and participation outcomes. The mediation analyses were conducted using PROCESS v. 2.16 (87), in line with current recommendations in the literature (85, 88, 89).

## Results

The multiple regression analysis predicting participation outcomes on the PART-O included 225 participants. In combination, the variables in the model accounted for a significant 38% of the variability in participation outcomes, *F*_(13, 211)_ = 9.93, *p* < 0.05, *R*^2^ = 0.38, adjusted *R*^2^ = 0.34.

Review of coefficients revealed that resilience made a unique contribution and was a significant predictor of participation outcomes (β = 0.17, *p* < 0.05). Furthermore, age at interview (β = −0.24, p < 0.01), premorbid IQ (β = 0.21, *p* < 0.05), PTA duration (β = −0.20, *p* < 0.01), total years of education (β = 0.18, *p* < 0.05), and years since injury (β = 0.11, *p* < 0.01) significantly predicted participation outcomes. The raw (*B*) and standardized (β) regression coefficients of the predictors, together with their squared semi-partial correlations indicating the unique variance predicted by each independent variable, are shown in Table 2. Participants' participation scores increased with higher resilience, higher years of education, higher premorbid IQ, greater years since injury, shorter PTA duration, and younger age. Gender, limb injury, employment status pre-injury, cognitive performance on the RAVLT, HADS anxiety and HADS depression, and family functioning were not significantly associated with participation outcomes.

**Table 2:** Unstandardized and standardized regression coefficients predicting participation outcomes and squared semi-partial correlations (*N* = 225).

**Variable**	**B (SE-B)**	**β**	**sr^2^**
**DEMOGRAPHIC VARIABLES**			
Age at interview	−0.008 (0.024)	−0.240^**^	0.04
Gender	−0.041 (0.069)	−0.035	0.00
Premorbid IQ	0.015 (0.005)	0.217^**^	0.03
Total years education	0.032 (0.011)	0.186^**^	0.02
Pre-injury employment	−0.003 (0.094)	−0.002	0.00
**INJURY VARIABLES**			
PTA	−0.003 (0.001)	−0.203^**^	0.04
Limb injury	0.003 (0.024)	0.007	0.00
Years since Injury	0.020 (0.010)	0.116^*^	0.04
RAVLT Trials	0.003 (0.003)	0.087	0.00
**POST-INJURY PERSONAL AND ENVIRONMENTAL VARIABLES**			
Resilience	0.012 (0.006)	0.173^*^	0.01
Depression	−0.015 (0.011)	−0.118	0.00
Anxiety	−0.001 (0.010)	−0.008	0.00
Family functioning	0.075 (0.056)	−0.085	0.00

*The dependent variable was Participation outcomes. R^2^ = 0.38, Adjusted R^2^ = 0.34. SE-B = standard error of unstandarised beta; sr^2^ = the squared semi-partial correlation indicating the unique variance predicted by each independent variable; PTA, Post traumatic amnesia; RAVLT Trials, Rey Auditory Verbal Learning Test Trials 1-5*.

* *p < 0.05*.

** *p < 0.01*.

**Figure 1:**
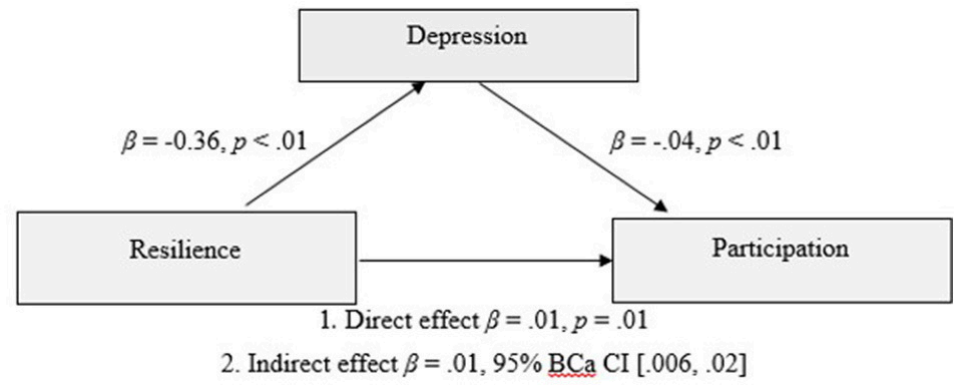
Model of resilience as a predictor of participation outcomes, mediated by depression scores of the participant.

The finding that depression and anxiety were not uniquely significant predictors of participation outcomes was unexpected. Given the association of resilience with depression and stress in previous studies (45, 53–56), as well as the findings of previous studies that depression and anxiety were associated with participation outcomes (10, 12, 31–33), it was considered important to further investigate their role, as potential mediators. Indeed, depression scores had medium to strong correlations with both participation (*r* = −0.39, *p* < 0.01) and resilience (−0.64, *p* < 0.01; See Table 3 for correlations). Anxiety had a weak correlation with participation (*r* = −0.28, *p* < 0.01), but also had a strong correlation with resilience (*r* = −0.62, *p* < 0.01). In order to assess whether depression mediated the relationship between resilience and participation outcomes, PROCESS v. 2.16 (87) was used with the default setting of 1000 bootstrapped samples on a model that included 243 participants. The relationship between resilience and participation outcomes was significant, *R* = 0.37, *R*^2^ = 0.13, *F*_(1, 241)_ = 37.15, *p* < 0.01. The inclusion of depression in the model representing the relationship between resilience and participation outcomes was also significant, *R* = 0.42, *R*^2^ = 0.18, *F*_(2, 240)_ = 26.30, *p* < 0.01. The indirect effect of resilience on participation outcomes via depression was significant β = 0.01, 95%, BCa CI [0.006, 0.02]. Refer to Figure 1.

**Table 3:** Correlations between demographic, injury, cognitive, personal, and participation variables.

**Variable**	**1**	**2**	**3**	**4**	**5**	**6**	**7**	**8**	**9**	**10**	**11**	**12**	**13**	**14**
1. Participation	–													
2. Limb injury	−0.09	–												
3. Age	−0.16^**^	−0.06	–											
4. Gender	−0.02	−0.08	0.11^*^	–										
5.Total years education	0.36^**^	−0.03	−0.01	0.06	–									
6. Pre-injury employment	0.04	−0.03	−0.13^*^	−0.27^**^	0.04^**^	–								
7. PTA	−0.17^**^	0.03	−0.12^*^	−0.09	0.06	0.11^*^	–							
8. RAVLT Trials 1-5	0.19^**^	−0.04	0.32^**^	0.20^**^	0.23^**^	−0.03	−0.15^*^	–						
9. Pre-morbid IQ	0.31^**^	−0.21^**^	0.24^**^	0.08	0.48^**^	0.03	0.05	0.37^**^	–					
10. Years since injury	0.23^**^	−0.17^**^	−0.05	0.02	0.02	0.05	−0.05	0.05	0.11^*^	–				
11. Resilience	0.34^**^	−0.08	0.00	−0.08	0.09	−0.00	0.00	0.02	0.06	0.17^**^	–			
12. Depression	−0.39^**^	0.13^*^	0.02	0.09	−0.24^**^	−0.05	−0.02	−0.19^**^	−0.12^*^	−0.24^**^	−0.64^**^	–		
13. Anxiety	−0.28^**^	0.11^*^	−0.08	0.12^*^	−0.19^**^	−0.08	−0.06	−0.09	−0.13^*^	−0.14^*^	−0.63^**^	0.65^**^	–	
14. Family functioning	−0.29^**^	0.08	0.09	−0.05	−0.16^*^	0.000	0.02	−0.03	−0.08	−0.07	−0.38^**^	0.45^**^	0.37^**^	–

*PTA, Post traumatic amnesia; RAVLT Trials, Rey Auditory Verbal Learning Test Trials 1-5*.

* *p < 0.05*;

** *p < 0.01*.

**Figure 2:**
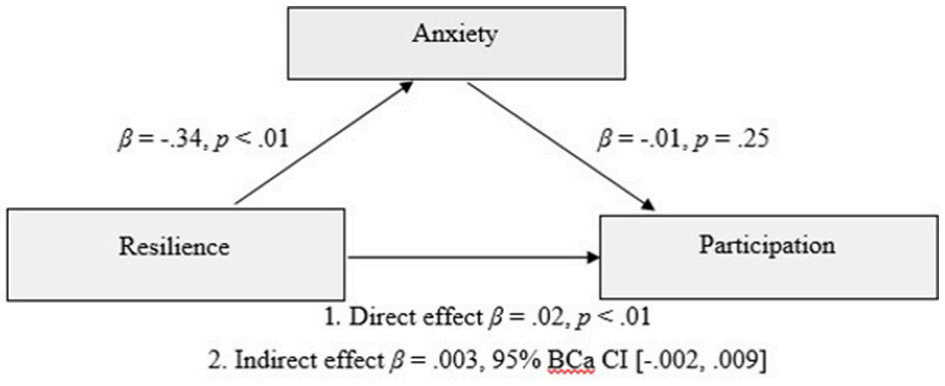
Model of resilience as a predictor of participation outcomes, mediated by anxiety scores of the participant.

PROCESS v. 2.16 (87) was also used to assess whether anxiety was a mediator of the relationship between resilience and participation outcomes (*n* = 244). The relationship between resilience and participation outcomes was significant, *R* = 0.37, *R*^2^ = 0.13, *F*_(1, 242)_ = 37.41, *p* < 0.01. The inclusion of anxiety in the model representing the relationship between resilience and participation outcomes was also significant, *R* = 0.37, *R*^2^ = 0.14, *F*_(2, 241)_ = 19.38, *p* < 0.01. However, the indirect effect of resilience on participation outcomes via anxiety was not significant β = 0.003, 95%, BCa CI [−0.002,0.009]. Refer to Figure 2.

## Discussion

The current study explored the association of resilience, alongside demographic, injury-related, emotional and environmental factors, with participation outcomes following TBI. A broad view of participation was taken, including social relationships and leisure activities, as well as productivity. The hypothesis that resilience would make an independent contribution to participation outcomes was supported, in a model that collectively predicted 38% of the variance in participation. In combination, higher resilience, higher years of education, higher premorbid IQ, greater time since injury, shorter PTA duration, and younger age, significantly predicted increased participation. Mediation analyses demonstrated that depression mediated the relationship between resilience and participation, however, anxiety was not a significant mediator of this relationship.

Resilience made a unique, albeit modest, contribution to participation outcome an average of 4 years and up to 13 years post injury. This suggests that despite the presence of significant and persisting disabilities in this mild to severe sample, personal qualities such as resilience may impact on the individual's capacity to reintegrate into the community. This confirms previous research finding an association between resilience and participation following TBI (39), and extends current knowledge by demonstrating this association beyond 12 months post-injury. Survivors with more resilient profiles also showed better emotional adjustment, which is consistent with previous research in similar samples (39, 44, 45). Exploratory mediation analyses demonstrated that depression was a significant mediator of the relationship between resilience and participation outcomes. Higher resilience may be a protective factor against depression which in turn affects levels of participation. Further, given the average time since injury was 5 years, this study suggests that the protective influence of resilience on depression may not be limited to the acute post injury period. Anxiety was not a statistically significant mediator of the relationship between resilience and participation. The reason for this finding is unclear but suggests that symptoms of depression and anxiety may interact somewhat differently with participation outcomes in individuals with TBI. Some support for this notion can be inferred from studies which have found employment outcomes to be associated with depression but not anxiety in TBI samples (90, 91). Further, given findings from previous research showing the association between resilience and anxiety is somewhat tempered by time post injury (39), it is also possible that any mediating effect of anxiety may be restricted to the acute post injury period.

There has been only one published study of a resilience-based intervention in the context of TBI (92, 93). This study examined the effectiveness of a psychoeducational and skill-building intervention, the “Resilience and Adjustment Intervention,” using a two-arm, parallel, randomized, controlled trial. Individuals who received the intervention showed a significant increase in resilience, however, this was not maintained at 3-months follow-up. It is important to acknowledge that resilience based interventions inherently assume that resilience is a construct that is modifiable and amenable to intervention, however, it remains debatable whether resilience may in fact be a stable trait.

The efficacy of interventions may also be impacted by cognitive impairments, which impede the capacity to take in, remember and follow through with the effects of therapy (45–47). Nevertheless, recent studies have demonstrated that individuals with TBI can benefit from psychological therapy that is adapted for their cognitive impairments (86). Moreover, TBI-specific treatment plans focused on building psychological strengths such as resilience early in the rehabilitation process, could potentially serve to circumvent the development of anxiety and depression and thereby enhance outcomes. Screening for resilience early in the rehabilitation process could also be used to identify individuals at risk of negative emotional responses. Screening processes may be further refined by previous research in this area, which has shown lower levels of resilience post TBI to be associated with being unemployed pre-injury, a lower level of education, being unmarried, being of minority race and having greater levels of disability (39).

These findings further highlight the importance of using multivariate models to identify the complex range of factors that combine to impact on outcome (24, 36–38, 94). The fact that they accounted for a relatively modest amount of variance, may reflect the complexity of the participation construct, including productivity, out and about/leisure, and social relations. Indeed, multivariate models specially examining employment outcomes have found that PTA, age, pre-injury employment, and physical, cognitive, and behavioral disability have predicted 60% or more of the variance in employment outcomes post-TBI (4, 12, 18, 19, 21). It is plausible that certain variables may be more strongly related to certain domains than others. However, as all three domains are inter-related and impact survivors' well-being and quality of life, it is important to study them in combination.

Of the demographic variables, age, pre-morbid IQ, and education contributed significantly to the prediction of participation. Consistent with previous research, younger individuals with higher IQ and higher education were found to have higher participation (10, 14, 22, 28, 29, 95–98). It is possible that increased participation with younger age may be related to improved mobility and physical capabilities in youth compared to the elderly (22, 28, 99, 100). It is also plausible that effects of normal aging may have also contributed to the lower PART-O scores, given older individuals are less likely to be engaged in work or study (10). Future research using a matched control sample would be of benefit to examine the trajectory in scores with normal aging. Higher education has been associated with better outcomes post TBI in previous research, possibly demonstrating the impact of cognitive reserve (101, 102). The cognitive reserve hypothesis postulates that individual differences in cognitive processes or neural networks allow some people to cope better with pathology from disease or brain damage (103). Higher education has been identified as a key source of cognitive reserve (102).

Duration of PTA emerged as the most significant predictor of participation, of the injury-related variables, ahead of current memory performance on RAVLT. This is consistent with previous research showing PTA duration to be a significant predictor of various outcome variables, including return to employment, functional independence, independent living, and cognitive function (14, 18–21, 97, 104). Considering that the average years post-injury in the study sample was 5 years, and extended up to 13 years, our results demonstrate that PTA remains a strong predictor of outcome even many years post-injury. Of the other injury-related variables, after controlling for injury severity, greater time since injury was associated with increased participation, as well as higher resilience, lower depression, and lower anxiety. These findings are consistent with longitudinal data showing that depression and anxiety decline gradually after peaking at 12 months post injury (105). Our findings contrast, however, with recent longitudinal data showing a decline in resilience with greater time post moderate to severe TBI (39). However, given that study was restricted to the first 12 months post injury, it is possible that levels of resilience may decrease in the first year after injury as survivors are confronted with numerous physical, cognitive and emotional challenges, but may, begin to increase over time alongside increased self-awareness, adaptive skill development, acquisition of coping skills, and psychological adjustment. Indeed, such processes of adaptation have been shown to continue over many years after injury (12, 106). Finally, limb injury was not a significant predictor, likely because the time elapsed since injury had allowed for recovery. Consistent with this, previous research has found limb injury to be a significant predictor of outcome at one year post injury, but not 5 years post injury (107).

Almost half of the sample reported unhealthy levels of family functioning. Although not a significant predictor of participation in the model, family functioning showed moderate correlations with resilience, depression and anxiety, and participation. This suggests an interplay between personal psychological strength and family support, and is consistent with previous research (44, 108–110). It is unknown whether healthy family functioning enhances an individual's resilience or whether resilient individuals are more satisfied within their family network. It may be the case that injured individuals with higher resilience are received better within the family unit, thus allowing for healthier family functioning. Further research would be of value to clarify how family functioning and resilience may interrelate, and how this may be associated with participation outcomes.

Notwithstanding the significance of identifying resilience as a potential predictor of participation outcomes post-TBI, the current study has certain limitations. It is possible that the sample was biased toward individuals who are generally inclined to be more participatory than others. This sample was also more highly educated than patients admitted for rehabilitation during the same period, which has implications for generalizability. The design of the study was cross-sectional and cannot be used to infer causation. A longitudinal study would be optimal for investigating resilience in conjunction with changes in emotional adjustment and participation outcomes over time in individuals with varying levels of injury severity. A longitudinal study design would also allow researchers to examine the trajectory of resilience over time, and factors that may contribute to resilience, such as psychosocial interventions, improved family support, return to work or increased status at work, development of friendship or intimate relationships. The amount of variance accounted for by the model was modest, suggesting many other variables not examined in this study may also contribute to participation outcomes. Finally, the measure of resilience used for the current study has yet to be fully validated. However, the TBI-QoL resilience scale was developed exclusively for individuals with TBI to address TBI-specific issues that generic measures fail to address (75) and thus was considered most appropriate for the current study.

In conclusion, this is the first large-scale study to examine the association between resilience and participation in a sample of individuals more than 12 months post mild to severe TBI. Whilst most previous predictive studies have focused on return to work, this study took a broader view of participation, using the PART-O as a measure encompassing engagement in social and leisure activities as well as productivity, which represent important contributors to an individual's well-being. The contribution of resilience to the model, although modest, highlights the significance of the person's response to injury. Given that most previous studies of resilience following TBI have focused on mild injuries only, this study extends previous research by demonstrating that, across the full spectrum of injury severity, and even in the presence of significant and persisting disabilities including cognitive impairments, personal qualities such as resilience can impact on the individual's capacity to reintegrate into the community many years post injury. Further, the additional finding that depression mediated the relationship between resilience and participation, suggests that resilience likely influences the probability of developing depressive symptoms, which in turn impacts on participation. Understanding the role of personal factors such as resilience has the potential to create a foundation for treatments that may foster optimistic and hopeful approaches after injury of any severity and enhance long-term survivor participation in society.

## Author contributions

JP, MS, CW, and AH contributed to the conception and design of this study. AH and staff from Monash-Epworth Rehabilitation Research Centre collected all data. CW conducted statistical analyses and lead the drafting of the manuscript. AH and JP provided ongoing supervision and consultation for CW. The interpretation of results and content of the discussion was led by CW, in collaboration JP and AH. All authors contributed to editing and reviewing of the manuscript.

### Conflict of interest statement

The authors declare that the research was conducted in the absence of any commercial or financial relationships that could be construed as a potential conflict of interest.
